# The first evidence of bovine viral diarrhea virus circulation in Libya

**DOI:** 10.14202/vetworld.2024.1012-1016

**Published:** 2024-05-09

**Authors:** Hania Elkhoja, Imad Buishi, Emiliana Brocchi, Santina Grazioli, Abdusalam Mahmoud, Ibrahim Eldaghayes, Abdunaser Dayhum

**Affiliations:** 1 National Center of Animal Health, Tripoli, Libya; 2 Department of Preventive Medicine, Faculty of Veterinary Medicine, University of Tripoli, Tripoli, Libya; 3 Istituto Zooprofilattico Sperimentale della Lombardia e dell’Emilia Romagna (IZSLER), Brescia, Italy; 4 Department of Microbiology and Parasitology, Faculty of Veterinary Medicine, University of Tripoli, Tripoli, Libya

**Keywords:** bovine viral diarrhea virus, Libya, risk factors, seroprevalence

## Abstract

**Background and Aim:**

Bovine viral diarrhea (BVD) is endemic in North Africa and the Mediterranean Basin with high socioeconomic impacts. However, there are no data on this disease in Libya. One of the aims of this study was to provide data on BVD in Libya, to fill in the gap in the region and to investigate the level of seroprevalence of BVD virus (BVDV) in Libya and associated risk factors.

**Material and Methods:**

A total of 1599 serum samples were collected from cattle herds belonging to seven Libyan regions. All sera were assayed using a screening enzyme-linked immunosorbent assay for the detection of antibodies against BVDV.

**Results:**

The overall seroprevalence of BVDV was estimated to be 48.6% (95% confidence interval, 46.08%–50.98%). A seroprevalence rate of 36.8% was detected in cattle aged <1 year, 41.0% in cattle aged between 1 and 2 years, and 49.7% in cattle aged >2 years. Statistically significant differences (p = 0.001) were observed between age groups. BVDV seroprevalence was significantly associated with geographical region (p = 0.033).

**Conclusion:**

To the best of our knowledge, this is the first study on BVD in Libya, and the results suggest that BVD is endemic in Libya. Further studies are required to isolate and characterize the circulated BVDV in Libya.

## Introduction

Bovine viral diarrhea (BVD) is a highly contagious disease that has been reported in many domestic and wildlife animals and is well known to be spread in many countries. However, BVD mainly affects cattle and can cause huge losses for cattle owners [1]. BVD is caused by Flaviviridae BVD virus (BVDV), which belongs to the genus *Pestivirus* [2, 3]. As documented by the International Committee on Taxonomy of Viruses, the *Pestivirus* genus has four species: BVDV-1, BVDV-2, classical swine fever virus, and border disease virus [2]. Two species of BVDV (BVDV-1 and BVDV-2) have been named because of their genetic and antigenic properties [4, 5]. The first description of BVDV dates back to 1949 in New York City [6, 7]. However, another study suggests that BVDV has been circulating for a long time in cattle populations [8]. The clinical manifestations of BVDV range from subclinical to severe, with high mortality rates. Clinical signs include gastrointestinal disorders and respiratory and reproductive symptoms. The clinical characteristics of BVDV infection vary among animal populations; accordingly, the type of infection depends on multiple factors, including the infecting viral strain, age, reproductive status, and immunological status of the infected animal [9]. The type of viral infection may be transient infection or persistent infection (PI).

Libya is located in the North of Africa, where the Mediterranean Sea lies to the north of the country and is bordered by six countries: Tunisia, Algeria, Niger, Chad, Sudan, and Egypt. Many diseases are known to spread between these countries due to uncontrolled and illegal movement of animals [10]. There is a lack of comprehensive research on the prevalence and impact of BVDV in North Africa. However, research on BVDV in North Africa is limited, and the available studies suggest that BVDV is a serious concern for the cattle industry in the region. The high prevalence of BVDV antibodies in Algerian cattle [11], the higher prevalence in dairy cattle in Tunisia [12], and the risk factors identified in Moroccan cattle [13] highlight the need for further research and effective management strategies to prevent and control BVDV in North Africa. In 1972, the first BVDV sample was isolated from a calf suffering from severe enteritis, as most of the reports came from Egypt, with very few reports showing the subtypes of circulating BVDV in animal herds.

In Algeria, Ait-Oudhia *et al*. [11] found a high prevalence of BVDV antibodies in cattle, with 53.8% of the animals testing positive. In another study, the prevalence was approximately 59.9% [14]. In Tunisia, Sassi *et al*. [12] reported a lower prevalence of BVDV antibodies in cattle, with 11.8% of animals testing positive. This study found that the prevalence of BVDV was higher in cattle raised for milk production than in cattle raised for meat production. Similarly, Fassi-Fihri *et al*. [13] found a moderate prevalence of BVDV antibodies in cattle in Morocco, with 33.3% of animals testing positive.

Many transboundary animal diseases (TADs) have been reported in Libya during the past few years, and instability in Libya has made it difficult to implement surveillance and monitoring programs for emerging and re-emerging infectious diseases with significant public health and socioeconomic impacts [10]. However, high commercial livestock activities in the country, especially in the hotspot (risky) areas where illegal animals cross the border from historically infected countries, make these areas pathways and portals of entrance to many TADs and increase the risk of emerging and re-emerging infectious diseases on a regional level [15–17]. In Libya, transitional animal movement is not well controlled between cities, and the extensive trade of livestock on a regional level increases the potential risk of the introduction of infected animals from neighboring endemic regions [18, 19]. In addition, there is a lack of official reports and historical data on epidemic outbreaks in the most investigated farms, which makes it difficult to predict how long the disease has spread throughout the country. The surveillance system and disease monitoring program need to be improved; therefore, the epidemiological patterns of BVDV in Japan are not well defined.

Because no previous study has been conducted on BVDV in Libya, epidemiological data on BVDV are very scarce. Therefore, the aim of this study was to estimate the seroprevalence of BVDV in cattle in Libya and to determine the risk factors associated with BVDV infection.

## Materials and Methods

### Ethical approval

This study was approved by the Department of Microbiology and Parasitology at the Faculty of Veterinary Medicine, University of Tripoli. The Ethical Committee at the National Center for Animal Health in Libya approved the sample collection (NCAH-15-2019).

### Study period and location

This study was conducted from January to December 2020 in seven Libyan regions. Table-1 shows the estimated total number of cattle in each region.

**Table 1:** Distribution of cattle in seven Libyan regions.

No.	Region	Cattle population
1	Green mountain	88,000
2	Benghazi	13,000
3	Middle area	2,000
4	Zawia	17,000
5	Tripoli	29,000
6	West mountain	1,000
7	Sabha	4,000
Total		154,000

### Sample collection and questionnaire survey

The sample size was determined based on the following equation:


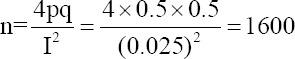


Where: p: expected prevalence; q: 1-p and l: allowed accepted error.

An android application named “Statistics and Sample Size Pro” was used to calculate the sampling size: https://play.google.com/store/apps/details?id=thaithanhtruc.info.sass&hl=en&gl=US.

Ten animals of different ages from each farm were selected. If there are less than 10 animals on the farm, all animals will be tested. There were 197 cattle farms. A total of 1599 serum samples were randomly collected from cattle herds belonging to seven Libyan regions. A structured well-designated questionnaire was used to collect all relevant data regarding the risk factors associated with infection (age group, sex, and region).

### Samples processing

Blood samples were collected aseptically from the jugular vein of the animals using sterile vacutainer tubes without anticoagulants. Approximately 10 mL of blood was collected from each animal. After collection, the labeled vacutainer tubes were transported in an icebox to the laboratory of the Libyan National Center of Animal Health (NCAH) in Tripoli. In the laboratory, the blood samples were processed to extract the serum. This was done by centrifuging the vacutainer tubes at 1,000× *g* for 10 min. The resulting serum was separated and the serum samples were stored at –20°C.

The collected serum samples were then shipped to Brescia, Italy, to the Istituto Zooprofilattico Sperimentale della Lombardia e dell’Emilia Romagna (IZSLER), a World Organization of Animal Health/Food and Agriculture Organization reference laboratory, and tested using enzyme-linked immunosorbent assay (ELISA) for the detection of antibodies against BVDV.

### Statistical analysis

For each age group, the prevalence and 95% confidence intervals (CIs) were calculated using the Bayesian approach of beta distribution. Univariate analysis was performed using the chi-square test to determine the association between outcome variables, including the status of BVDV infection and risk factors. In addition, the OR was used to estimate the effect size as the association between the seroprevalence of BVDV and potential risk factors was analyzed using logistic regression. p < 0.05 was considered statistically significant.

## Results

Overall seroprevalence of BVDV was estimated to be 48.6% (95% CI: 46.08%–50.98%) in this study. The results of the univariate analysis of independent variables showed statistically significant differences (p = 0.05, Table-2).

**Table 2:** The univariant analysis of BVDV seroprevalence and associated risk factors.

Risk factors	Animal tested	Animal affected (%)	DF	X^2^	p-value
Sex	1599	48.5	1	8.41	**0.004**
Male	198	38.9			
Female	1401	49.9			
Region			6	13.67	**0.033**
Green Mountain	478	49.1			
Benghazi	106	56.6			
Middle area	55	58.2			
Zawiyah	268	44.4			
Tripoli	519	49.1			
West Mountain	136	47.8			
Sabha	37	45.0			
Age group			2	12.94	**0.001**
<1 year	185	36.76			
1–2 year	605	41			
>2 year	809	48.6			

BVDV=Bovine viral diarrhea vírus, Bold p-value indicates significant differences.

**Figure-1:**
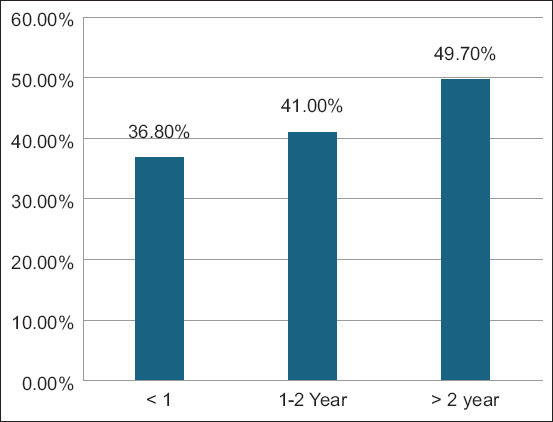
The seroprevalence of bovine viral diarrhea virus according to age groups.

High seroprevalence of BVDV was reported in age groups **<** 1 year 36.76% (95% CI, 29.81%–43.70%), 1–2 years 41% (95% CI, 37.07%–44.91%), and >2 years 48.6% (95% CI, 45.13%–52.02%) (Figure-1). The seroprevalence of BVDV was found to be significantly higher (p = 0.001) in adult animals than in young animals.

**Figure-2:**
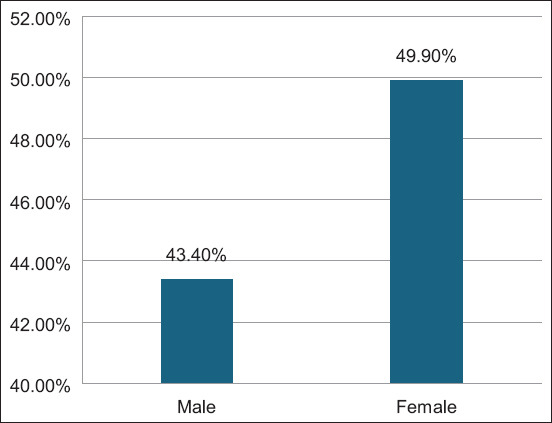
Seroprevalence of bovine viral diarrhea virus according to animal sex.

BVDV seroprevalence in males and females was high at 38.9% (95% CI: 32.10%–45.68%) and 49.9% (95% CI: 47.27%–52.51%), respectively (Figure-2). Significantly (p = 0.004), the seroprevalence of BVDV among cattle was influenced by gender.

**Figure-3:**
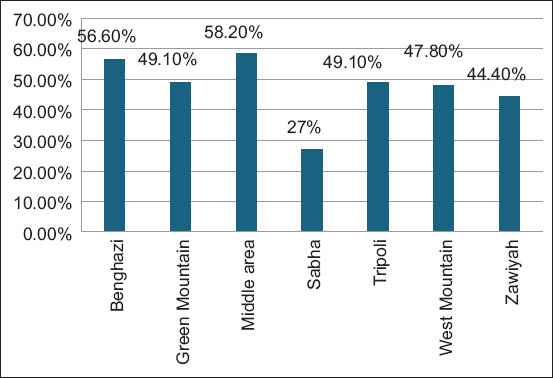
The seroprevalence of bovine viral diarrhea virus according to the Libyan geographical regions.

The highest seroprevalence of BVDV was observed in the middle area (58.2%; 95% CI, 45.15%–71.22%) and the lowest seroprevalence was observed in Sabha (Southern region) (27.03%; 95% CI, 12.72%–41.34%), followed by the Zawiyh region (44.4%; 95% CI, 38.45%–50.35%) (Figure-3). BVDV seroprevalence was significantly associated with geographical region (p = 0.033).

## Discussion

The BVD is well known, described, and documented in a wide range of literature that preserves almost North Africa and the Mediterranean region. However, this study is the first to investigate BVDV among non-vaccinated cattle populations in Libya. As expected, BVDV seroprevalence among dairy cattle in different parts of the country was high in this study. Several studies have reported a high seroprevalence of BVDV in North Africa and Mediterranean regions [20–22]. In line with other similar studies indicating different seroprevalence between males and females with higher seroprevalence in females, the significant difference between sexes (p = 0.004) found in this study could be explained by the fact that fewer males were present in cattle herds than females. Herd’s men sell bulls after weaning, resulting in a higher number of older females than older males. It was estimated that the high seroprevalence of BVDV is higher in adult animals than in young animals. These results are in agreement with several studies that reported frequent higher seroprevalence in adults [23, 24]. Comparatively, the relatively high (36.76%) seroprevalence of BVDV among young animals in the present study is evidence of the constant endemicity of BVD within dairy cattle herds in the country. A significant association between BVDV infection and newborn animals immunotolerant to PI and BVDV is in agreement with several studies [25, 26] from different parts of the world. Younger animals (calf) play a crucial role in transmission and PI within bovine herds with BVDV [27, 28]. PI animals shed high BVDV titers from nasal and ocular secretions, urine, semen, colostrum/milk, and feces [29, 30]. Unfortunately, our study was not designed to determine PI among pregnant dams and offspring calves in cattle populations. Therefore, it remains unclear whether PI calf could play a significant role in the epidemiology of BVDV in Libyan cattle populations. In addition, another question arises as to the impact of BVDV on the reproductive performance of dairy cattle in Libya.

The present study reported the highest BVDV seroprevalence in the middle, Benghazi, and Green Mountain regions, followed by the Western, Zawiyah, and Tripoli regions (Figure-3). Comparatively, the lowest BVDV seroprevalence was reported in Sabha (the southern region). Differences in seroprevalence values among regions may be attributable to (influenced by) animal dynamics, density, animal housing system, and distribution of cattle at the national regional level (herd size per farm; Table-1). Uncontrolled animal movement, nomadism, and animal trade pathways (market) are potential factors that affect the spatial distribution of BVDV among dairy cattle in different Libyan regions. The high seroprevalence rate indicates that BVD is constantly endemic in almost all Libyan regions studied. Despite significant differences (p = 0.033) in BVDV seroprevalence at the geographical level, seroprevalence in almost all Libyan regions was somewhat uniformly distributed. The highest seroprevalence values reported at the national and regional levels indicate a wide spatial distribution of BVDV infection in most dairy cow populations. These high seroprevalence values of BVDV indicate the endemicity of the disease and PI of BVDV among cattle herds, potentially impacting the country’s dairy farming industry. Therefore, it is necessary to implement biosecurity measures, first by implementing strict quarantine measures for exotic animal trade and second by introducing a vaccination policy against BVDV. In addition, continuous testing (monitoring) of BVD carriers (PI) to prevent the infection of vaccinated herds and isolation of the reactor is required.

## Conclusion

This study showed that BVDV infection is widespread in Libyan cattle. The results of this study suggest that BVD is endemic in Libya, with constant exposure of animals to BVD during their life. More studies are still needed, and the Libyan National Center for Animal Health should consider introducing vaccination against BVD as a control strategy in Libya.

## Data Availability

The supplementary data can be available from the corresponding authors upon a reasonable request.

## Authors’ Contributions

HE, IB, IE, and AD: Conceptualization; HE, IB, EB, SG, AM, IE, and AD: Methodology; IB, AM, IE, and AD: Validation; HE, IB, EB, SG, AM, and AD: Formal analysis; HE, IB, AM, IE, and AD: Investigation; AM, IE, and AD: Data curation; HE, IE, AM, and AD: Writing–original draft preparation; HE, IB, AM, IE, and AD: Writing–review and editing; IB and AD: Supervision. All authors have read, reviewed, and approved the final manuscript.
